# Predictive Modeling in Race Walking

**DOI:** 10.1155/2015/735060

**Published:** 2015-08-03

**Authors:** Krzysztof Wiktorowicz, Krzysztof Przednowek, Lesław Lassota, Tomasz Krzeszowski

**Affiliations:** ^1^Faculty of Electrical and Computer Engineering, Rzeszów University of Technology, 35-959 Rzeszów, Poland; ^2^Faculty of Physical Education, University of Rzeszów, 35-959 Rzeszów, Poland

## Abstract

This paper presents the use of linear and nonlinear multivariable models as tools to support training process of race walkers. These models are calculated using data collected from race walkers' training events and they are used to predict the result over a 3 km race based on training loads. The material consists of 122 training plans for 21 athletes. In order to choose the best model leave-one-out cross-validation method is used. The main contribution of the paper is to propose the nonlinear modifications for linear models in order to achieve smaller prediction error. It is shown that the best model is a modified LASSO regression with quadratic terms in the nonlinear part. This model has the smallest prediction error and simplified structure by eliminating some of the predictors.

## 1. Introduction

The level of today's high-performance sport is very high and very even. Both coaches and competitors are forced to search for and use newer and sometimes innovative solutions in the process of sports training [1]. A solution supporting this process may be the application of various types of regression models.

Prediction in sport concerns many aspects including the prediction of performance results [2, 3] or predicting sporting talent [4, 5]. Models predicting results in sport, taking into account the seasonal statistics of each team, were also constructed [6]. The application of predictive models in athletics was described by Maszczyk et al. [2], where the regression was used to predict results in a javelin throw. These models were applied to support the choice and selection of prospective javelin throwers.

Prediction of sports results using linear regression was also presented in the work by Przednowek and Wiktorowicz [7]. A linear predictive model, implemented by ridge regression, was applied to predict the outcomes of a walking race after the immediate preparation phase. As input for the model, the basic somatic features (height and weight) and training loads (training components) for each day of training were provided, and the output was the result expected over a distance of 5 km. In addition to linear models, artificial neural networks, whose parameters were specified in cross-validation, were also used to implement this task.

In the paper by Drake and James [8], the regressions estimating the results over distances of 5, 10, 20, and 50 km and the levels of the selected physiological parameters (e.g., VO_2_max) were presented. The regressions applied were the classical linear models, and the *R* ^2^ criterion was chosen for the quality evaluation. This study included 23 women and 45 men. The amount of collected data was different depending on the task and ranged from 21 to 68 records.

A nonlinear regression equation to predict the maximum aerobic capacity of footballers was proposed by Chatterjee et al. [9]. The data came from 35 young players aged from 14 to 16. The experiment was to verify the use of the 20 m MST (Multistage Shuttle Run Test) in evaluating the performance of VO_2_max. The talent of young hockey players was identified by Roczniok et al. [5] using a regression equation. The research involved 60 boys aged between 15 and 16, who attended selection camps. The applied regression model classified candidates for future training, based on selected parameters of the players. The logistic regression was used in the model as the classification method.

The nonlinear predictive models used in sport are also based on the selected methods of “data mining” [10]. Among them, an important role is played by fuzzy logic expert systems. Papić et al. [4] described practical application of such a system. The proposed system was based on knowledge of experts in the field of sport, as well as the data obtained as a result of motor tests. The model suggested the most suitable sport and it was designed to search for prospective sports talents.

The application of fuzzy modeling techniques in sports prediction was also presented by Mężyk and Unold [11]. The goal of their paper was to find the rules that can express swimmer's feelings the day after in-water training. The data was collected for two months among competitors practicing swimming. The swimmers were characterized by a good level of sports attainment (2nd sport class). The material obtained consisted of 12 attributes, and the total number of models was 480, out of which 136 were used in the final stage. The authors proved that their method was characterized by better predictive ability than the traditional methods of classification.

Other papers also concern the use of artificial neural networks in sports prediction [6]. Neural models are used to analyze the effectiveness of the training of swimmers, to identify handball players' tactics, or to predict sporting talent [12]. Many studies present the application of neural networks in various aspects of sports training [13–15]. These models support the planning of training loads, practice control, or the selection of sports.

An approach developed by the authors is the construction of models performing the task of predicting the results achieved by a competitor in the proposed sports training. This allows for the proper selection of training components and thus supports the achievement of the desired result. The aim of this study is to determine the effectiveness of selected linear and nonlinear models in predicting the outcome in a 3-kilometer walking race for the proposed training. The research hypothesis of the paper is stated as follows: the prediction error of 3 kilometers' result in race walking for nonlinear models can be smaller than for linear models.

The paper is organized as follows. In Section 2, the training data of the race walkers recorded during annual training cycle is described.  Section 3 contains the methods used to build the linear and nonlinear predictive models, including ordinary least squares regression, regularized methods, that is, ridge, LASSO, and elastic net regressions, nonlinear least squares regression, and artificial neural networks as multilayer perceptron and radial basis function network. In Section 3, the criterion used to evaluate the performance of the models, calculated using mean square error in the process of cross-validation, is also defined.  Section 4 describes the procedures used for building models and their evaluation in *R* language and STATISTICA software. The obtained results are analyzed and discussed in Section 5. Finally, in Section 6, the performed work is concluded.

## 2. Material

The predictive models were built using the training data of athletes practising race walking. The analysis involved a group of colts and juniors from Poland. Among the competitors were the finalists in the Polish Junior Indoor Championships and the Polish Junior Championships. The data of race walkers was recorded during the 2011-2012 season in the form of training means and training loads. The training mean is the type of work performed while the training load is the amount of work at a particular intensity done by an athlete during exercise [1]. In the material, which has been collected, 11 means of training were distinguished. The material was drawn from the annual training cycle for the following four phases: transition, general preparation, special preparation, and starting phase. The training data has the form of sums of training loads completed in one month of the chosen training phase. The material included 122 training patterns made by 21 race walkers.

Control of the training process in race walking requires different tests of physical fitness at every training level. Because this research concerns the competitors in colt and junior categories, thus in order to determine a unified criterion of the level of training, a result for 3000 m race walking was used. The choice of the distance of 3000 m is valid because this is the indoor walking competition.

The description of the variables under consideration and their basic statistics are presented in Table 1. The variables are as follows: arithmetic mean of $\overset{-}{x}$, minimum value *x*_min_, maximum value *x*_max_, standard deviation SD, and coefficient of variation $V=SD/\overset{-}{x}\cdot 100\%$. The qualitative variables are *X*_1_, *X*_2_, *X*_3_, *X*_4_, which take their values from the set {0,1}. The other variables, that is, *X*_5_,…, *X*_18_, are quantitative variables. If the value at inputs *X*_1_, *X*_2_, *X*_3_ is 0, it means that the transitional period is considered. Setting the value 1 on one of the inputs *X*_1_, *X*_2_, *X*_3_, it means the training period is selected. The variable *X*_4_ represents the gender of the competitor, where the value 0 denotes a female, while the value 1 denotes a male, and the age is represented by *X*_5_. Basic somatic features of race walkers such as weight and height are presented in the form of BMI (*X*_6_) expressed by the formula

(1) $$\begin{array}{c} \text{BMI}=\frac{M}{H^{2}}\;\left[\;kg/m^{2}\;\right], \end{array}$$

where *M* is the body weight [kg] and *H* is the body height [m]. The variable *X*_7_ denotes the current result over 3 km in seconds. Training loads are characterized by the following variables: running exercises (*X*_8_), walking with different levels of intensity (*X*_9_, *X*_10_, *X*_11_), exercises forming different types of endurance (*X*_12_, *X*_13_, *X*_14_), exercises forming techniques (*X*_15_), exercises forming muscle strength (*X*_16_), exercises forming general fitness (*X*_17_), and warming up exercises (*X*_18_).

An example of data used for building the model has the form

(2) x5=0,1,0,0,23,22.09,800,32,400,112,20,16,32.4,48,8,280,640,400,y5=800.

The vector **x**_5_ represents a 23-year-old race walker with BMI = 22.09 kg/m^2^, who completes training in the special preparation phase. The result both before and after the training was the same and is equal to 800 s.

## 3. Methods

In this study, two approaches were considered. The first approach was based on white box models realized by modern regularized methods. These models are interpretable because their structure and parameters are known. The second approach was based on black box models realized by artificial neural networks.

### 3.1. Constructing Regression Models

**Figure 1:**
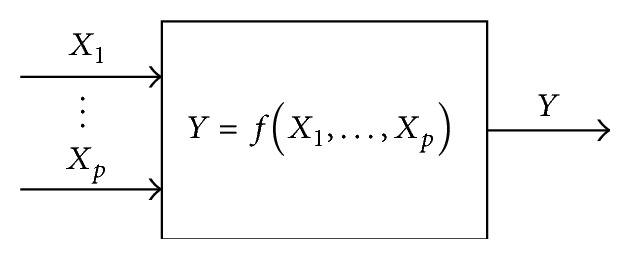
A diagram of a system with multiple inputs and one output.

Consider a multivariable regression model with the inputs (*predictors* or* regressors*)  *X*_*j*_,  *j* = 1,…, *p*, and one output (*response*) *Y* shown in Figure 1. We assume that the model is linear and has the form

(3) Y^=w0+X1w1+⋯+Xpwp=w0+∑j=1pXjwj,

where $\hat{Y}$ is the estimated response and *w*_0_, *w*_*j*_ are unknown weights of the model. The weight *w*_0_ is called* constant term* or* intercept*. Furthermore, we assume that the data is standardized and centered and the model can be simplified to the form (see, e.g., [16])

(4) Y^=X1w1+⋯+Xpwp=∑j=1pXjwj.

Observations are written as pairs (**x**_*i*_, *y*_*i*_), where **x**_*i*_ = [*x*_*i*1_,…, *x*_*ip*_],  *i* = 1,…, *n*, *x*_*ij*_ is the value of the *j*th predictor in the *i*th observation, and *y*_*i*_ is the value of the response in the *i*th observation. Based on formula (4), the *i*th observation can be expressed as

(5) y^ixi1w1+⋯+xipwp∑j=1pxijwj=xiw,

where **w** = [*w*_1_,…, *w*_*p*_]^*T*^. Introducing matrix **X** in the form of

(6) $$\begin{array}{c} X=\left[\begin{array}{cccc} x_{11} & x_{12} & \cdots & x_{1p}\\ x_{21} & x_{22} & \cdots & x_{2p}\\ \vdots & \vdots & \ddots & \vdots \\ x_{n1} & x_{n2} & \cdots & x_{np} \end{array}\right] \end{array}$$

formula (5) can be written as

(7) $$\begin{array}{c} \hat{y}=Xw, \end{array}$$

where $\hat{y}={\left[{\hat{y}}_{1},\ldots ,{\hat{y}}_{n}\right]}^{T}$.

In order to construct regression models, an error (residual) is introduced as the difference between the real value *y*_*i*_ and the estimated value ${\hat{y}}_{i}$ in the form of

(8) $$\begin{array}{c} e_{i}=y_{i}-{\hat{y}}_{i}=y_{i}-\overset{p}{\underset{j=1}{\sum }}x_{ij}w_{j}=y_{i}-x_{i}w. \end{array}$$

Using matrix (6), the error can be written as

(9) $$\begin{array}{c} e=y-\hat{y}=y-Xw, \end{array}$$

where **e** = [*e*_1_,…, *e*_*n*_]^*T*^ and  **y** = [*y*_1_,…, *y*_*n*_]^*T*^.

Denoting by *J*(**w**, ·) the* cost function*, the problem of finding the optimal estimator can be formulated as to minimize the function *J*(**w**, ·), which means solving the problem

(10) $$\begin{array}{c} \hat{w}=\underset{w}{argmin}\left(\;J\left(\;w,\cdot \;\right)\;\right), \end{array}$$

where $\hat{w}$ is the vector of solutions.

Depending on the function *J*(**w**, ·), different regression models can be obtained. In this paper, the following models are considered: ordinary least squares regression (OLS), ridge regression, LASSO (least absolute shrinkage and selection operator), elastic net regression (ENET), and nonlinear least squares regression (NLS).

### 3.2. Linear Regressions

In OLS regression (see, e.g., [16–18]) the model is calculated by minimizing the sum of squared errors:

(11) Jw=eTe=y−XwTy−Xw=y−Xw22,

where ‖·‖_2_ denotes the Euclidean norm (*L*_2_). Minimizing the cost function (11), which is the quadratic function of **w**, we get the following solution:

(12) $$\begin{array}{c} \hat{w}={\left(\;X^{T}X\;\right)}^{-1}X^{T}y. \end{array}$$

It should be noted that solution (12) does not exist if the matrix **X** ^*T*^ **X** is singular (due to correlated predictors or if *p* > *n*). In this case, different methods of regularization, including the previously mentioned ridge, LASSO, and elastic net regressions, can be used.

In ridge regression by Hoerl and Kennard [19], the cost function includes a penalty and has the form

(13) Jw,λ=eTe+λwTw=y−XwTy−Xw+λwTw=y−Xw22+λw22.

The parameter *λ* ≥ 0 determines the size of the penalty: for *λ* > 0, the model is penalized, for *λ* = 0, ridge regression reduces to OLS regression. Solving problem (10) for ridge regression, we get

(14) $$\begin{array}{c} \hat{w}={\left(\;X^{T}X+\lambda I\;\right)}^{-1}X^{T}y, \end{array}$$

where **I** is the identity matrix with the size of *p* × *p*. Because the diagonal of the matrix **X** ^*T*^ **X** is increased by a positive constant, the matrix **X** ^*T*^ **X** + *λ* **I** is invertible and the problem becomes nonsingular.

In LASSO regression by Tibshirani [20], similarly to ridge regression, the penalty is added to the cost function, where the *L*_1_-norm (the sum of absolute values) is used:

(15) Jw,λ=eTe+λzTw=y−XwTy−Xw+λzTw=y−Xw22+λw1,

where **z** = [*z*_1_,…, *z*_*p*_]^*T*^,  *z*_*j*_ = sgn(*w*_*j*_), and ‖·‖_1_ denotes the Manhattan norm (*L*_1_). Because problem (10) is not linear in relation to **y** (due to the use of *L*_1_-norm), the solution cannot be obtained in the compact form as in ridge regression. The most popular algorithm used in this case is the LARS algorithm (least angle regression) by Efron et al. [21].

In elastic net regression by Zou and Hastie [22], the features of ridge and LASSO regressions are combined. The cost function in the so-called* naive elastic net* has the form of

(16) Jw,λ1,λ2=eTe+λ1zTw+λ2wTw=y−XwTy−Xw+λ1zTw+λ2wTw=y−Xw22+λ1w1+λ2w22.

To solve the problem, Zou and Hastie [22] proposed the LARS-EN algorithm, which was based on the LARS algorithm developed for LASSO regression. They used the fact that elastic net regression reduces to LASSO regression for the augmented data set (**X** ^*∗*^, **y** ^*∗*^).

### 3.3. Nonlinear Regressions

To take into account the nonlinearity in the models, we can apply the transformation of predictors or use nonlinear regression. In this paper, the latter solution is applied.

In OLS regression, the model is described by formula (5), while in more general nonlinear regression the relationship between the output and the predictors is expressed by a certain nonlinear function *f*(·) in the form of

(17) $$\begin{array}{c} {\hat{y}}_{i}=f\left(\;x_{i},w\;\right). \end{array}$$

In this case, the cost function *J*(**w**) is formulated as

(18) Jw∑i=1nei2=∑i=1nyi−y^i2∑i=1nyi−fxi,w2.

Since the minimization of function (18) is associated with solving nonlinear equations, numerical optimization is used in this case. The main problem connected with the construction of nonlinear models is the choice of the appropriate function *f*(·).

### 3.4. Artificial Neural Networks

Artificial neural networks (ANNs) were also used for building predictive models. Two types of ANNs were implemented: a multilayer perceptron (MLP) and networks with radial basis function (RBF) [18].

The MLP network is the most common type of neural models. The calculation of the output in 3-layer multiple-input-one-output network is performed in* feed-forward* architecture. In the first step, *m* linear combinations, or the so-called* activations*, of the input variables are constructed as

(19) $$\begin{array}{c} a_{k}=\overset{p}{\underset{j=1}{\sum }}x_{j}w_{kj}^{\left(1\right)}, \end{array}$$

where *k* = 1,…, *m* and *w*_*kj*_^(1)^ denotes the weights for the first layer. From the activations *a*_*k*_, using a nonlinear* activation function h*(·), hidden variables *z*_*k*_ are calculated as

(20) $$\begin{array}{c} z_{k}=h\left(\;a_{k}\;\right). \end{array}$$

The function *h*(·) is usually chosen as logistic or “tanh” function. The hidden variables are used next to calculate the* output activation*

(21) $$\begin{array}{c} a=\overset{m}{\underset{k=1}{\sum }}z_{k}w_{k}^{\left(2\right)}, \end{array}$$

where *w*_*k*_^(2)^ are weights for the second layer. Finally, the output of the network is calculated using an activation function *σ*(·) in the form of

(22) $$\begin{array}{c} y=\sigma \left(\;a\;\right). \end{array}$$

For regression problems, the function *σ*(·) is chosen as identity function, and so we obtain *y* = *a*. The MLP network utilizes iterative supervised learning known as* error backpropagation* for training the weights. This method is based on* gradient descent* applied to the sum of squares function. To avoid the problem with* overtraining* the network, the number *m* of hidden neurons, which is a free parameter, should be determined to give the best predictive performance.

In the RBF network, the concept of radial basis function is used. Linear regression (5) is extended by linear combinations of nonlinear functions of the inputs in the form of

(23) $$\begin{array}{c} {\hat{y}}_{i}=\overset{p}{\underset{j=1}{\sum }}\phi_{j}\left(\;x_{ij}\;\right)w_{j}=\varphi \left(\;x_{i}\;\right)w, \end{array}$$

where **φ** = [*ϕ*_1_,…, *ϕ*_*p*_]^*T*^ is a vector of* basis functions*. Using nonlinear basis functions, we get a nonlinear model, which is, however, a linear function of parameters *w*_*j*_. In the RBF network, the hidden neurons perform a radial basis function whose value depends on the distance from selected center *c*_*j*_:

(24) $$\begin{array}{c} \varphi \left(\;x_{i},c\;\right)=\varphi \left(\;\left\|\;x_{i}-c\;\right\|\;\right), \end{array}$$

where **c** = [*c*_1_,…, *c*_*p*_] and ‖·‖ is usually the Euclidean norm. There are many possible choices for the basis functions, but the most popular is Gaussian function. It is known that RBF network can exactly interpolate any continuous function; that is, the function passes exactly through every data point. In this case, the number of hidden neurons is equal to the number of observations and the values of coefficients *w*_*j*_ are found by simple standard inversion technique. Such a network matches the data exactly, but it has poor predictive ability because the network is overtrained.

### 3.5. Choosing the Model

In this paper, the best predictive model is chosen using leave-one-out cross-validation (LOOCV) method [23], in which the number of tests is equal to the number of data *n* and one pair (**x**_*i*_, *y*_*i*_) creates a testing set. The quality of the model is evaluated by means of the square root of the mean square error (RMSE_CV_) defined as

(25) MSECV=1n∑i=1nyi−y^−i2,RMSECV=MSECV,

where ${\hat{y}}_{-i}$ denotes the output of the model built in the *i*th step of validation process using a data set containing no testing pair (**x**_*i*_, *y*_*i*_) and MSE_CV_ is the mean square error.

In order to describe the measure to which the model fits the training data, the root mean square error of training (RMSE_T_) is considered. This error is defined as

(26) MSET=1n∑i=1nyi−y^i2,RMSET=MSET,

where ${\hat{y}}_{i}$ denotes the output of the model built in the *i*th step using the full data set and MSE_T_ is the mean square error of training.

## 4. Implementation of the Predictive Models

All the regression models were calculated using *R* language with additional packages [24].

The lm.ridge function from “MASS” package [25] was used for calculating OLS regression (where *λ* = 0) and ridge regression (where *λ* > 0). With the function enet included in the package “elastic net” [26], LASSO regression and elastic net regression were calculated. The parameters of the enet function are *s* ∈ [0,1] and *λ* ≥ 0, where *s* is a fraction of the *L*_1_ norm, whereas *λ* denotes *λ*_2_ in formula (16). The parameterization of elastic net regression using the pair (*λ*, *s*) instead of (*λ*_1_, *λ*_2_) in formula (16) is possible because elastic net regression can be treated as LASSO regression for an augmented data set (**X** ^*∗*^, **y** ^*∗*^) [22]. Assuming that *λ* = 0, we get LASSO regression with one parameter *s* for the original data (**X**, **y**).

All the nonlinear regression models were calculated using the nls function coming from the “stats” package [27]. It calculates the parameters of the model using the nonlinear least squares method. One of the parameters of the nls function is a formula that specifies the function *f*(·) in model (18). To calculate the weights, Gauss-Newton's algorithm was used which was selected by default in the nls function. In all the calculations, it was assumed that the initial values of the weights are equal to zero.

For the implementation of artificial neural networks, StatSoft STATISTICA [28] software was used. The learning of MLP networks was implemented using the BFGS (Broyden-Fletcher-Goldfarb-Shanno) algorithm [18]. While calculating the RBF network, the parameters of the basis functions were automatically set by the learning procedure.

The parameters in all models were selected using leave-one-out cross-validation. In the case of regularized regressions, the penalty coefficients were calculated, while, in the case of neural networks, the number of neurons in the hidden layer was calculated. The primary performance criterion of the model was RMSE_CV_ error. Cross-validation functions in the STATISTICA program were implemented using Visual Basic language.

## 5. Results and Discussion

From a coach's point of view, the prediction of results is very important in the process of sport training. A coach using the model, which was constructed earlier, can predict how the training loads will influence the sport outcome. The presented models can be used for predictions based on the proposed monthly training introduced as the sum of the training loads of each type implemented in a given month.

The results of the research are presented in Table 2; the description of the selected regressions will be presented in the next paragraphs. Linear models such as OLS, ridge, and LASSO regressions have been calculated by the authors in work [3]. They will be briefly described here. The nonlinear models implemented using nonlinear regression and artificial neural networks will be discussed in greater detail. All the methods will be compared taking into account the accuracy of the prediction.

### 5.1. Linear Regressions

The regression model calculated by the OLS method generated the prediction error RMSE_CV_ = 26.90 s and the training error RMSE_T_ = 22.70 s (Table 2). In the second column of Table 2, the weights *w*_0_ and *w*_*j*_ are presented.

The search for the ridge regression model is based on finding the parameter *λ*, for which the model achieves the smallest prediction error. In this paper, ridge regression models for *λ* changing from 0 to 2 with step of 0.1 were analyzed. Based on the results, it was found that the best ridge model is achieved for *λ*_opt_ = 1. The prediction error RMSE_CV_ = 26.76 s was smaller than in the OLS model, while the training error RMSE_T_ = 22.82 s was greater (Table 2). The obtained ridge regression improved the predictive ability of the model. It is seen from Table 2 that as in the case of OLS regression, all weights are nonzero and all the input variables are used in computing the output of the model.

The LASSO regression model was calculated using the LARS-EN algorithm, in which the penalty is associated with the parameter *s* changing from 0 to 1 with step of 0.01. It was found that the optimal LASSO regression is calculated for *s*_opt_ = 0.78. The best LASSO model generates the error RMSE_CV_ = 26.20 s, which improves the results of OLS and ridge models. However, it should be noted that this model is characterized by the worst data fit with the greatest training error RMSE_T_ = 22.89 s. The LASSO method is also used for calculating an optimal set of input variables. It can be seen in the fourth column of Table 2 that the LASSO regression eliminated the five input variables (*X*_4_, *X*_6_, *X*_14_, *X*_15_, and *X*_17_), which made the model simpler than for OLS and ridge regression.

The use of elastic net regression model has not improved the value of the prediction error. The best model was obtained for a pair of parameters *s*_opt_ = 0.78 and *λ*_opt_ = 0. Because the parameter *λ* is zero, the model is identical to the LASSO regression (fourth column of Table 2).

### 5.2. Nonlinear Regressions

Nonlinear regression models were obtained using various functions *f*(·) in formula (18). It was assumed that the function *f*(·) consists of two components: the linear part, in which the weights are calculated as in OLS regression, and the nonlinear part containing expressions of higher orders in the form of a quadratic function of selected predictors:

(27) $$\begin{array}{c} f\left(\;x_{i},w,v\;\right)=\overset{p}{\underset{j=1}{\sum }}x_{ij}w_{j}+\overset{p}{\underset{j=1}{\sum }}x_{ij}^{2}v_{j}, \end{array}$$

where **w** = [*w*_1_,…, *w*_*p*_]^*T*^ is the vector of the weights of the linear part and **v** = [*v*_1_,…, *v*_*p*_]^*T*^ is the vector of the weights of the nonlinear part. The following cases of nonlinear regression were considered (Table 3), wherein each of the following models does not take into account the squares of qualitative variables *X*_1_, *X*_2_, *X*_3_, and *X*_4_ (*v*_1_ = *v*_2_ = *v*_3_ = *v*_4_ = 0):

(i) NLS1: both the weights of the linear part and the weights *v*_5_,…, *v*_18_ of the nonlinear part are calculated.
(ii) NLS2: the weights of the linear part are constant, and their values come from the OLS regression (the second column of Table 2); the weights *v*_5_,…, *v*_18_ of the nonlinear part are calculated (the third column of Table 3).
(iii) NLS3: the weights of the linear part are constant, and their values come from the ridge regression (the third column of Table 2); the weights *v*_5_,…, *v*_18_ of the nonlinear part are calculated (the fourth column of Table 3).
(iv) NLS4: the weights of the linear part are constant, and their values come from the LASSO regression (the fourth column of Table 2); the weights *v*_5_, *v*_7_,…, *v*_13_, *v*_16_, *v*_18_ of the nonlinear part are calculated (the fifth column of Table 3).

Based on the results shown in Table 3, the best nonlinear regression model is the NLS4 model, that is, the modified LASSO regression. This model is characterized by the smallest prediction error and the reduced number of predictors.

### 5.3. Neural Networks

**Figure 2:**
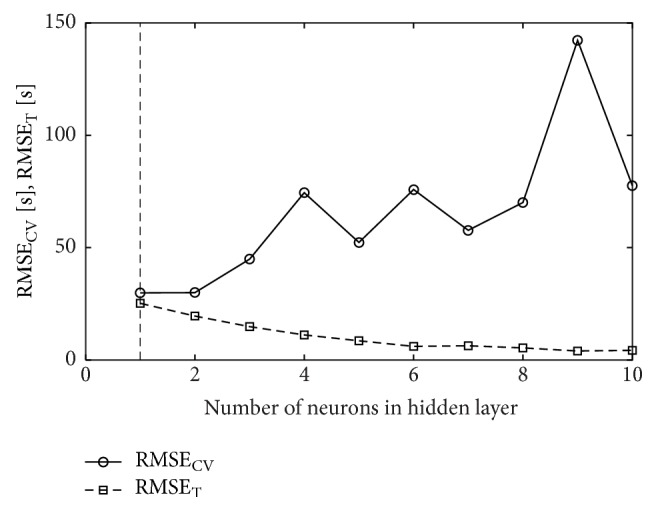
Cross-validation error (RMSE_CV_) and training error (RMSE_T_) for MLP(tanh) neural network; vertical line drawn for *m* = 1 signifies the number of hidden neurons chosen in cross-validation.

**Figure 3:**
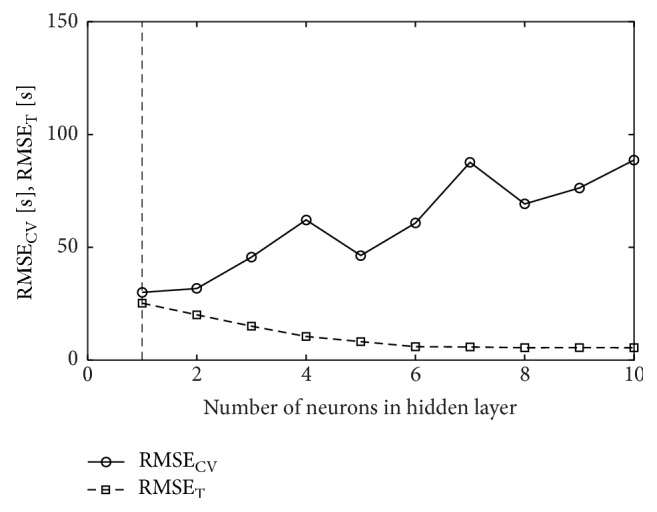
Cross-validation error (RMSE_CV_) and training error (RMSE_T_) for MLP(exp) neural network; vertical line drawn for *m* = 1 signifies the number of hidden neurons chosen in cross-validation.

**Figure 4:**
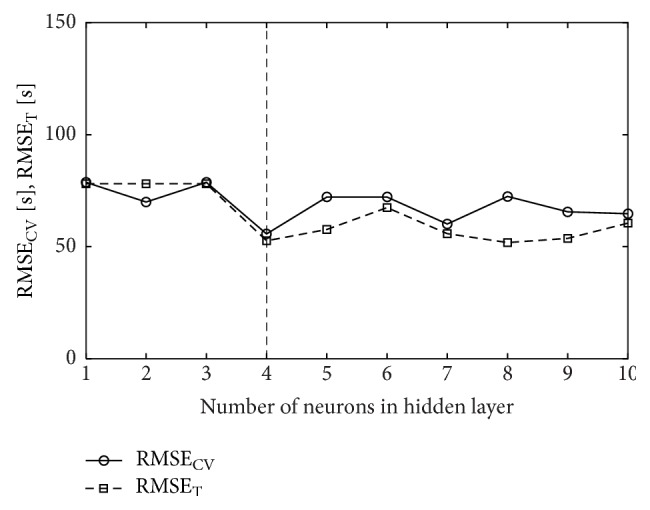
Cross-validation error (RMSE_CV_) and training error (RMSE_T_) for RBF neural network; vertical line drawn for *m* = 4 signifies the number of hidden neurons chosen in cross-validation.

In order to select the best structure of a neural network, the number of neurons *m* ∈ [1,10] in the hidden layer was analyzed. In Figures 2, 3, and 4, the relationships between cross-validation error and the number of hidden neurons are presented. The smallest cross-validation errors for the MLP(tanh) and MLP(exp) networks were obtained for one hidden neuron (18-1-1 architecture) and they were, respectively, 29.89 s and 30.02 s (Table 4). For the RBF network, the best architecture was the one with four neurons in the hidden layer (18-4-1) and cross-validation error in this case was 55.71 s. Comparing the results, it is seen that the best model is the MLP(tanh) network with the 18-1-1 architecture. However, it is worse than the best regression model NLS4 (Table 3) by more than 5 seconds.

## 6. Conclusions

This paper presents linear and nonlinear models used to predict sports results for race walkers. Introducing a monthly training schedule for a selected phase in the annual cycle, a decline in physical performance may be predicted based on the generated results. Thanks to that, it is possible to take into account earlier changes in the scheduled training. The novelty of this research is the use of nonlinear models, including modifications of linear regressions and artificial neural networks, in order to reduce the prediction error generated by linear models. The best model was the nonlinear modification of LASSO regression for which the error was 24.6 seconds. In addition, the method has simplified the structure of the model by eliminating 9 out of 32 predictors. The research hypothesis was confirmed. Comparing with other results is difficult because there is a lack of publications concerning predictive models in race walking.

Experts in the fields of sports theory and training were consulted during the construction of the models in order to maintain the theoretical and practical principles of sport training. The importance of the work is that practitioners (coaches) can use predictive models for planning of training loads in race walking.

## Figures and Tables

**Table 1:** The variables and their basic statistics.

Variable	Description	$\overset{-}{x}$	*x*_min_	*x*_max_	SD	*V* [%]
*Y*	Result over 3 km [s]	936.9	780	1155	78.4	8.4
*X*_1_	General preparation phase	—	—	—	—	—
*X*_2_	Special preparation phase	—	—	—	—	—
*X*_3_	Starting phase	—	—	—	—	—
*X*_4_	Competitor's gender	—	—	—	—	—
*X*_5_	Competitor's age [years]	18.9	14	24	3.0	15.6
*X*_6_	BMI (body mass index) [kg/m^2^]	19.3	16.4	22.1	1.7	8.7
*X*_7_	Current result over 3 km [s]	962.6	795	1210	87.7	9.1
*X*_8_	Overall running endurance [km]	30.9	0	56	10.6	34.4
*X*_9_	Overall walking endurance in the 1st intensity range [km]	224.6	57	440	96.1	42.8
*X*_10_	Overall walking endurance in the 2nd intensity range [km]	53.2	0	120	34.6	65.1
*X*_11_	Overall walking endurance in the 3rd intensity range [km]	7.9	0	30	9.4	119.7
*X*_12_	Short tempo endurance [km]	8.9	0	24	5	56.0
*X*_13_	Medium tempo endurance [km]	8.3	0	32.4	8.6	103.2
*X*_14_	Long tempo endurance [km]	12.9	0	56	16.1	125.0
*X*_15_	Exercises forming technique (rhythm) of walking [km]	4.4	0	12	4.2	96.0
*X*_16_	Exercises forming muscle strength [min]	90.2	0	360	104.8	116.3
*X*_17_	Exercises forming general fitness [min]	522.0	120	720	109.9	21.0
*X*_18_	Universal exercises (warm up) [min]	317.3	150	420	72.5	22.8

**Table 2:** Coefficients of linear models and linear part of nonlinear model NLS1 and error results.

Regression	OLS	RIDGE	LASSO, ENET	NLS1
*w*_0_	237.2	325.7	296.6	2005
*w*_1_	45.67	34.67	32.75	41.24
*w*_2_	90.61	74.84	71.91	77.12
*w*_3_	39.70	27.49	24.45	−3.439
*w*_4_	−2.838	2.424		15.45
*w*_5_	−0.9755	−1.770	−1.416	−22.44
*w*_6_	1.072	0.5391		−24.71
*w*_7_	0.7331	0.6805	0.7069	−1.782
*w*_8_	−0.2779	−0.3589	−0.3410	−1.500
*w*_9_	−0.1428	−0.1420	−0.1364	−0.0966
*w*_10_	−0.1579	−0.0948	−0.0200	0.7417
*w*_11_	0.7472	0.4352	0.0618	0.6933
*w*_12_	0.4845	0.3852	0.1793	−0.6726
*w*_13_	0.1216	0.1454	0.1183	−0.0936
*w*_14_	−0.1510	−0.0270		2.231
*w*_15_	−0.5125	−0.3070		0.7349
*w*_16_	−0.0601	−0.0571	−0.0652	−0.2685
*w*_17_	−0.0153	−0.0071		0.0358
*w*_18_	−0.0115	−0.0403	−0.0220	−0.0662

RMSE_CV_ [s]	26.90	26.76	26.20	28.83

RMSE_T_ [s]	22.70	22.82	22.89	20.21

**Table 3:** Coefficients of nonlinear part of nonlinear models and error results (all coefficients have to be multiplied by 10^−2^).

Regression	NLS1	NLS2	NLS3	NLS4
*v*_5_	53.35	−0.3751	−0.3686	−0.6995
*v*_6_	59.43	−1.0454	−1.3869	
*v*_7_	0.1218	0.0003	0.0004	0.0001
*v*_8_	1.880	0.0710	0.0372	−0.0172
*v*_9_	−0.0016	0.0093	0.0093	0.0085
*v*_10_	−0.6646	−0.0577	−0.0701	−0.1326
*v*_11_	−3.0394	−0.3608	0.0116	0.8915
*v*_12_	4.8741	0.3807	0.4170	1.0628
*v*_13_	0.4897	−0.2496	−0.2379	−0.1391
*v*_14_	−4.7399	−0.1141	−0.1362	
*v*_15_	−13.6418	1.3387	0.8183	
*v*_16_	0.0335	−0.0015	−0.0003	−0.0004
*v*_17_	−0.0033	−0.0006	−0.0006	
*v*_18_	0.0054	0.0012	0.0013	−0.0002

RMSE_CV_ [s]	28.83	25.24	25.34	**24.60**

RMSE_T_ [s]	20.21	22.63	22.74	22.79

**Table 4:** The number of hidden neurons and error results for the best neural nets.

ANN	MLP(tanh⁡)	MLP(exp⁡)	RBF
*m *	1	1	4

RMSE_CV_ [s]	**29.89**	30.02	55.71

RMSE_T_ [s]	25.19	25.17	52.63
